# Quantitative assessment of erector spinae muscles and prognosis in elderly patients with pneumonia

**DOI:** 10.1038/s41598-021-83995-3

**Published:** 2021-02-22

**Authors:** Hiroki Yoshikawa, Kosaku Komiya, Takashi Yamamoto, Naoko Fujita, Hiroaki Oka, Eiji Okabe, Mari Yamasue, Kenji Umeki, Bruce K. Rubin, Kazufumi Hiramatsu, Jun-ichi Kadota

**Affiliations:** 1 Department of Respiratory Medicine and Infectious Diseases, Oita University Faculty of Medicine, 1-1 Idaigaoka, Hasama-machi, Yufu, Oita 879-5593 Japan; 2 Department of Internal Medicine, Tenshindo Hetsugi Hospital, 5956 Nihongi, Nakahetsugi, Oita, Oita 879-7761 Japan; 3 Department of Pediatrics, Virginia Commonwealth University School of Medicine, 1217 East Marshall Street, KMSB, Room 215, Richmond, VA 23298 USA; 4 Department of Medical Safety Management, Oita University Faculty of Medicine, 1-1 Idaigaoka, Hasama-machi, Yufu, Oita 879-5593 Japan; 5 Nagasaki Harbor Medical Center, 6-39 Shinchi-machi, Nagasaki, 850-8555 Japan

**Keywords:** Medical research, Clinical trial design, Clinical trials

## Abstract

Erector spinae muscle (ESM) size has been reported as a predictor of prognosis in patients with some respiratory diseases. This study aimed to assess the association of ESM size on all-cause in-hospital mortality among elderly patients with pneumonia. We retrospectively included patients (age: ≥ 65 years) admitted to hospital from January 2015 to December 2017 for community-acquired pneumonia who underwent chest computed tomography (CT) on admission. The cross-sectional area of the ESM (ESMcsa) was measured on a single-slice CT image at the end of the 12th thoracic vertebra and adjusted by body surface area (BSA). Cox proportional hazards regression models were used to assess the influence of ESMcsa/BSA on in-hospital mortality. Among 736 patients who were admitted for pneumonia, 702 patients (95%) underwent chest CT. Of those, 689 patients (98%) for whom height and weight were measured to calculate BSA were included in this study. Patients in the non-survivor group were significantly older, had a greater frequency of respiratory failure, loss of consciousness, lower body mass index, hemoglobin, albumin, and ESMcsa/BSA. Multivariate analysis showed that a lower ESMcsa/BSA independently predicted in-hospital mortality after adjusting for these variables. In elderly patients with pneumonia, quantification of ESMcsa/BSA may be associated with in-hospital mortality.

## Introduction

Many high- and middle-income countries are facing an aging society. This trend in the demographics has increased the prevalence of age-related diseases including pneumonia^1^. Pneumonia in elderly individuals is often caused by aspiration of oral secretions, as a result of weakness and swallowing dysfunction^2,3^. Dysphagia, malnutrition, and cerebrovascular diseases, have been reported to be associated with pneumonia^4^. These factors have also been associated with the prognosis in elderly patients with pneumonia^5,6^. Zhang et al. showed that prognostic factors for mortality varied among patients with pneumonia in different age groups^7^. Male sex, cancer, and congestive heart failure are associated with mortality among patients aged 65–84 years, and altered mental status, tachycardia, blood urea nitrogen, hypoxemia, arterial pH, and pleural effusion are associated with mortality in patients aged > 85 years.

Pneumonia is classified as community-acquired pneumonia (CAP) or hospital-acquired pneumonia. In Japan chest computed tomography (CT) is often used to evaluate hospitalized patients with pneumonia^8^. The CT measurement of erector spine muscles (ESM) or pectoralis muscles has been associated with severity and prognosis in patients with chronic obstructive pulmonary disease^9–12^, idiopathic pulmonary fibrosis^13–17^, and *Mycobacterium avium* complex (MAC) lung disease^18,19^. The size of the thoracic skeletal muscles as a surrogate for muscle strength and nutrition may be associated with disease severity or progression.

Pneumonia among the elderly is primary due to swallowing dysfunction and decreased physical activity. Therefore, we hypothesized that the size of the ESM, would predict mortality in elderly patients with pneumonia. ESM area is an objective measure of muscle mass. Although low BMI predicted mortality in these elderly subjects, fat, bone, and muscle all contribute to BMI measurement. A low ESMcsa/BSA was an independent mortality risk although it is not clear if decreased muscle mass predisposes to increased risk (e.g. through weak cough, ineffective ventilation, and aspiration) of if debilitation is a common factor leading to both decreased muscle mass and increased morbidity and mortality.

## Results

### Baseline characteristics

**Figure 1:**
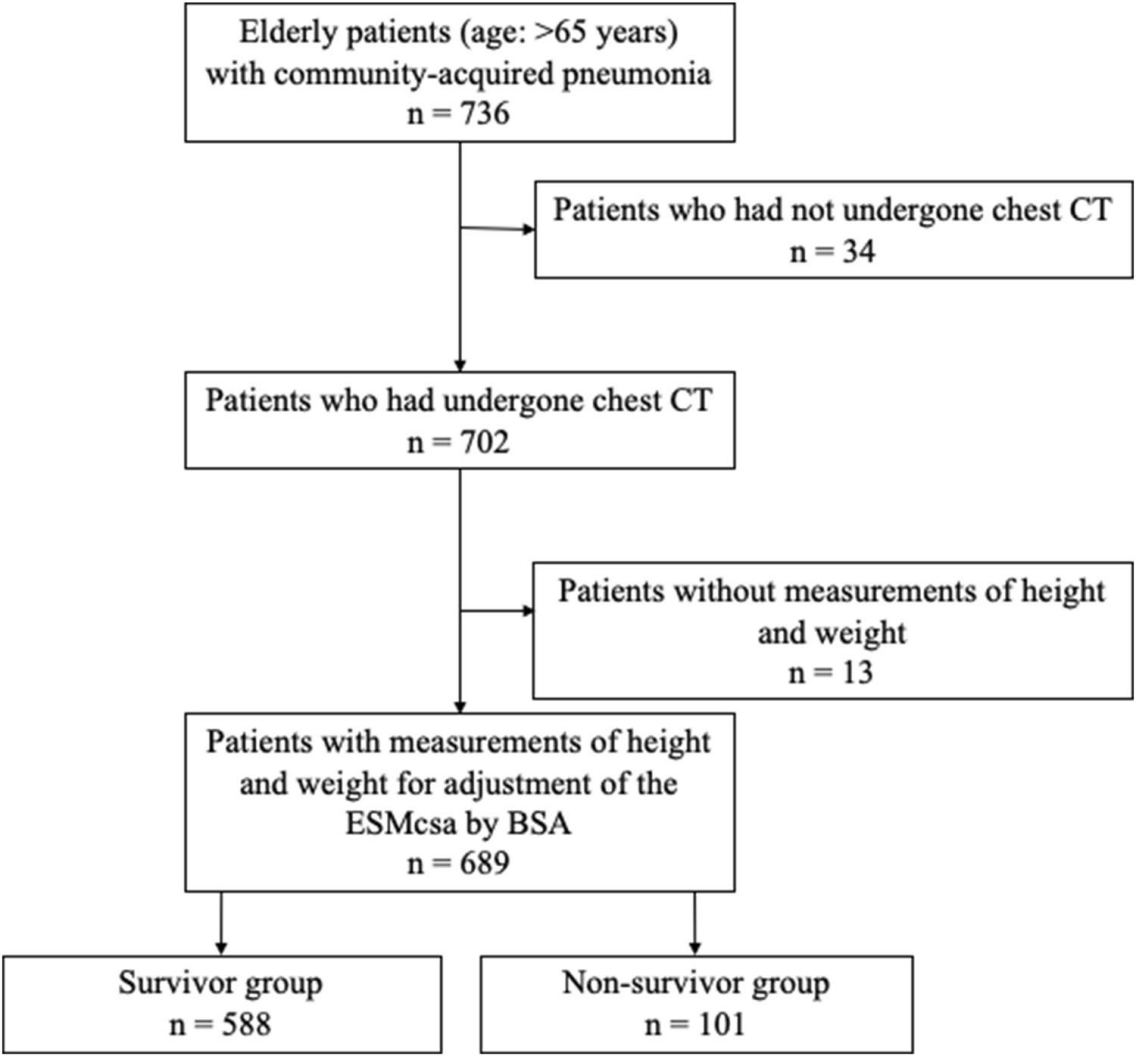
A flow chart of the patient charts evaluated for this study and the number of patients in each group.

In total, 736 elderly patients were admitted to our institute during the study period, and 702 patients (95%) had a chest CT scan. Of those, 689 (98%) who had their height and weight measured on admission were eventually included in this study (Fig. 1). Among them, 101 patients (15%) died in the hospital. The median hospital duration in survivors was 21 days (interquartile range [IQR] 12–43 days) and the median survival in patients who died was 28 days (IQR 6–48 days). Pathogens were isolated in 288 of 698 patients (41%): methicillin‐resistant *Staphylococcus aureus* (n = 65) followed by *Klebsiella pneumoniae* (n = 49), *Pseudomonas aeruginosa* (n = 43), *Streptococcus pneumoniae* (n = 42), *Haemophilus influenzae* (n = 23), methicillin-susceptible *Staphylococcus aureus* (n = 20), *Moraxella catarrhalis* (n = 17), *Escherichia coli* (n = 14), *Enterobacter spp.* (n = 8), *Haemophilus parainfluenzae* (n = 7), *Klebsiella oxytoca* (n = 6), *Legionella spp.* (n = 4), *Prevotella* (n = 4).

Other baseline characteristics, including ESMcsa/BSA, are presented in Table 1. The median age was > 80 years and almost half of the patients were admitted from nursing homes. The Barthel Index on admission was lower than that obtained before admission.

**Table 1:** Baseline characteristics of patients.

Variables	All cases N = 689
Female	329 (48)
Age, years	84.7 ± 8.0
BMI, kg/m^2^	19.5 ± 4.0
Loss of consciousness	77 (11)
Systolic blood pressure, mmHg	124 ± 26
Respiratory failure	288 (42)
Nursing home residency	341 (49)
Current smoker	9 (1)
Former smoker	248 (36)
The Barthel Index before admission	46 ± 43
The Barthel Index on admission	23 ± 30
Pneumococcal vaccination within 5 years	135 (20)
Chronic obstructive pulmonary disease	105 (15)
Chronic heart failure	135 (20)
Cerebrovascular diseases	233 (34)
Diabetes mellitus	108 (16)
Dementia	226 (33)
Gastroesophageal reflux	15 (2)
Malignancies	36 (5)
Corticosteroids	48 (7)
White blood cells, 10^3^/µL	10.7 ± 5.1
Hemoglobin, g/dL	11.8 ± 4.5
C-reactive protein, mg/dL	8.9 ± 7.4
Aspartate aminotransferase, IU/L	31.1 ± 69.4
Urea nitrogen, mg/dL	24.3 ± 15.4
Creatinine, mg/dL	1.04 ± 0.84
Albumin, g/dL	3.2 ± 2.0
ESMcsa, cm^2^	19.7 ± 7.6
ESMcsa/BSA	14.4 ± 5.0
Inappropriate use of antibiotics	89 (13)
Duration of hospital stay	34 ± 36

Data are presented as the number (%) or mean ± SD.

*BMI* body mass index, *BSA* body surface area, *ESM* erector spinae muscle, *ESMcsa* cross-sectional area of ESM, *n* number, *SD* standard deviation.

### Bivariate correlation analysis

Using Spearman’s test, the ESMcsa/BSA values were significantly correlated with the following consecutive variables: age (*ρ* = − 0.104, p = 0.006), BMI (*ρ* = 0.225, p < 0.001), the Barthel Index before admission (*ρ* = 0.252, p < 0.001), the Barthel Index on admission (*ρ* = 0.231, p < 0.001), hemoglobin levels (*ρ* = 0.111, p = 0.004), and albumin levels (*ρ* = 0.203, p < 0.001). In contrast, according to Pearson’s correlation test, these values were weakly correlated only with the Barthel Index obtained before admission (*r* = 0.099, p = 0.009).

### Analysis of in-hospital and 30-day mortality

As shown in Table 2, those patients who died in-hospital were significantly older, more had respiratory failure, loss of consciousness, chronic heart failure, malignancies, lower BMI, hemoglobin levels, albumin levels, and Barthel Index on admission. Use of corticosteroids did not affect mortality in patients with pneumonia. The ESMcsa/BSA value was significantly lower in those who died in-hospital than in survivors. There was no significant difference in inappropriate use of antibiotics between survivors and non-survivors. As shown by Model 1 and Model 2 (Table 3), multiple Cox proportional hazards analyses showed that a lower ESMcsa/BSA value independently predicted in-hospital mortality. A lower BMI, respiratory failure, malignancies, and lower albumin levels were also significantly associated with in-hospital mortality.

**Table 2:** Univariate Cox proportional hazards analyses of all-cause in-hospital mortality.

	Survivor group n = 588	Non-survivor group n = 101	HR (95% CI)	p
Female	290 (49)	39 (39)	0.711 (0.474–1.064)	0.097
Age, years	84.2 ± 8.1	87.5 ± 6.8	1.045 (1.017–1.075)	0.002
BMI, kg/m^2^	19.8 ± 4.0	17.8 ± 3.7	0.912 (0.862–0.965)	0.001
Loss of consciousness	53 (9)	24 (24)	2.116 (1.333–3.360)	0.001
Systolic blood pressure, mmHg	125 ± 25	121 ± 30	0.997 (0.989–1.005)	0.481
Respiratory failure	201 (34)	66 (65)	2.385 (1.573–3.615)	< 0.001
Nursing home residency	272 (46)	70 (69)	1.974 (1.284–3.034)	0.002
Current smoker	9 (2)	0 (0)	0.049 (0.000–1,399.931)	0.565
Former smoker	222 (38)	38 (38)	1.023 (0.683–1.534)	0.911
Barthel Index before admission	49 ± 43	27 ± 36	0.993 (0.987–0.998)	0.009
Barthel Index on admission	26 ± 31	7 ± 16	0.975 (0.962–0.988)	< 0.001
Pneumococcal vaccination within 5 years	115 (20)	20 (20)	1.063 (0.650–1.738)	0.809
Chronic obstructive pulmonary disease	89 (15)	16 (16)	0.804 (0.443–1.459)	0.474
Chronic heart failure	105 (18)	30 (30)	1.696 (1.104–2.604)	0.016
Cerebrovascular diseases	199 (34)	34 (34)	0.790 (0.520–1.202)	0.271
Diabetes mellitus	92 (16)	16 (16)	0.996 (0.583–1.701)	0.989
Dementia	181 (31)	45 (45)	1.261 (0.848–1.876)	0.252
Gastroesophageal reflux	13 (2)	2 (2)	0.834 (0.205–3.383)	0.799
Malignancies	23 (4)	13 (13)	2.528 (1.402–4.559)	0.002
Corticosteroids	42 (7)	6 (6)	0.823 (0.360–1.880)	0.644
White blood cells, 10^3^/µL	10.7 ± 5.0	10.9 ± 5.8	1.000 (1.000–1.000)	0.381
Hemoglobin, g/dL	11.9 ± 4.8	11.0 ± 2.3	0.907 (0.831–0.989)	0.028
C-reactive protein, mg/dL	8.7 ± 7.1	9.7 ± 8.6	1.010 (0.984–1.037)	0.440
Aspartate aminotransferase, IU/L	31.3 ± 74.4	30.1 ± 24.7	0.999 (0.994–1.004)	0.691
Urea nitrogen, mg/dL	23.6 ± 14.1	28.8 ± 21.0	1.009 (0.997–1.020)	0.128
Creatinine, mg/dL	1.02 ± 0.83	1.12 ± 0.89	1.108 (0.903–1.359)	0.325
Albumin, g/dL	3.4 ± 2.2	2.8 ± 0.6	0.403 (0.299–0.542)	< 0.001
ESMcsa, cm^2^	20.4 ± 7.6	15.2 ± 5.6	0.999 (0.999–0.999)	< 0.001
ESMcsa/BSA	14.9 ± 4.9	11.6 ± 4.1	0.870 (0.827–0.915)	< 0.001
Inappropriate use of antibiotics	73 (12)	16 (16)	1.104 (0.646–1.887)	0.717

Data are presented as the number (%) or mean ± SD.

*BMI* body mass index, *BSA* body surface area, *CI* confidence interval, *ESM* erector spinae muscle, *ESMcsa* cross-sectional area of ESM, *HR* hazard ratio, *SD* standard deviation.

**Table 3:** Multivariate Cox proportional hazards analyses of all-cause in-hospital mortality.

	Model 1	p	Model 2	p
	HR (95% CI)		HR (95% CI)	
Age, years	1.024 (0.991–1.057)	0.151	1.022 (0.990–1.056)	0.172
BMI, kg/m^2^	0.930 (0.873–0.990)	0.023	0.930 (0.874–0.990)	0.022
Loss of consciousness	1.384 (0.844–2.270)	0.197	1.226 (0.742–2.028)	0.427
Respiratory failure	2.207 (1.431–3.404)	< 0.001	2.083 (1.347–3.220)	0.001
Barthel Index before admission	0.998 (0.992–1.004)	0.484	–	–
Barthel Index on admission	–	–	0.985 (0.970–0.999)	0.040
Heart failure	1.523 (0.950–2.442)	0.081	1.554 (0.976–2.474)	0.063
Malignancies	3.208 (1.710–6.018)	< 0.001	3.338 (1.784–6.245)	< 0.001
Hemoglobin, g/dL	1.002 (0.958–1.049)	0.925	1.003 (0.961–1.048)	0.880
Albumin, g/dL	0.520 (0.357–0.757)	0.001	0.530 (0.364–0.771)	0.001
ESMcsa/BSA	0.864 (0.813–0.919)	< 0.001	0.866 (0.816–0.920)	< 0.001

Data are presented as the number (%) or mean ± SD.

*BMI* body mass index, *BSA* body surface area, *CI* confidence interval, *ESM* erector spinae muscle, *ESMcsa* cross-sectional area of ESM, *HR* hazard ratio, *SD* standard deviation.

**Figure 2:**
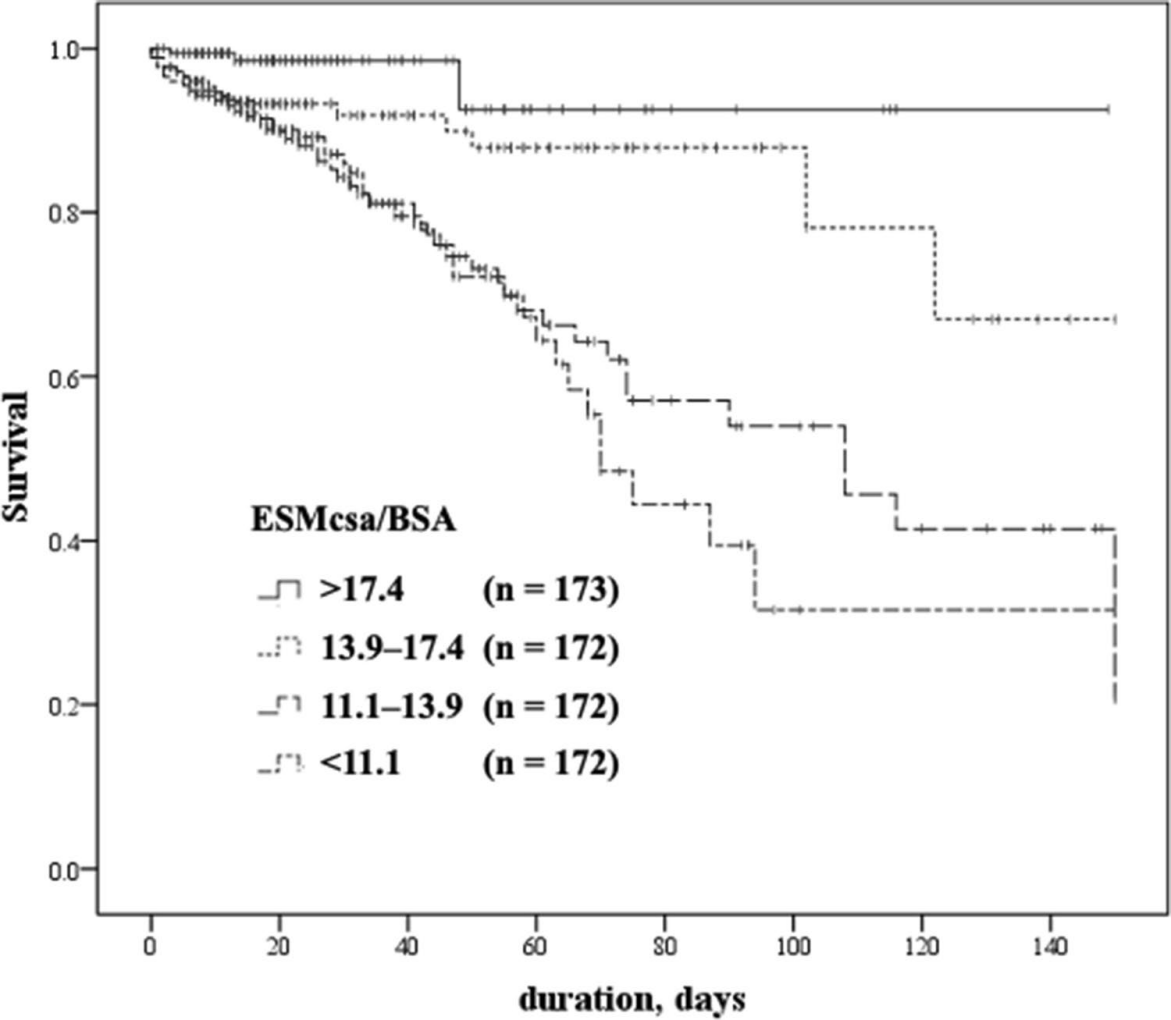
Kaplan–Meier curves stratified by the cross-sectional area of the erector spinae muscle (ESMcsa) adjusted by the body surface area (BSA). The cutoff values correspond to 75, 50, 25 percentile values (17.4, 13.9 and 11.1, respectively). Elderly patients with pneumonia with lower ESMcsa/BSA values exhibited significantly worse survival rates (p < 0.00; log-rank test).

Kaplan–Meier survival curves based on the ESMcsa/BSA subgroups with cutoff values (75, 50, and 25 percentile values of 17.3, 13.9, and 11.1, respectively) are illustrated in Fig. 2. According to the log-rank test, elderly patients with pneumonia with lower ESMcsa/BSA values had significantly worse survival (p < 0.001).

When 30-day mortality is used as outcome, patients in the non-survivor group were significantly older, more frequently male, had respiratory failure, loss of consciousness, malignancies, higher creatinine level, lower BMI, hemoglobin levels, albumin levels, and/or Barthel Index before and on admission as shown in Table 4. The ESMcsa/BSA value was significantly lower in those who died than the survivor group. As shown in Table 5, multiple Cox proportional hazards analyses showed that a lower ESMcsa/BSA value independently predicted 30-day mortality. Male, respiratory failure, malignancies, and a lower albumin level and Barthel Index on admission were also significantly associated with 30-day mortality.

**Table 4:** Univariate Cox proportional hazards analyses of all-cause 30-day mortality.

	Survivor group n = 635	Non-survivor group n = 54	HR (95% CI)	p
Female	310 (49)	19 (35)	0.571 (0.327–0.998)	0.049
Age, years	84.4 ± 8.0	88.3 ± 6.9	1.059 (1.019–1.101)	0.003
BMI, kg/m^2^	19.6 ± 4.0	17.8 ± 3.6	0.897 (0.829–0.970)	0.007
Loss of consciousness	62 (10)	15 (28)	3.103 (1.710–5.633)	< 0.001
Systolic blood pressure, mmHg	125 ± 25	122 ± 33	0.996 (0.985–1.007)	0.442
Respiratory failure	249 (39)	39 (72)	3.702 (2.031–6.750)	< 0.001
Nursing home residency	307 (48)	35 (65)	1.576 (0.900–2.762)	0.112
Current smoker	9 (1)	0 (0)	0.049 (0.000–4,172.427)	0.602
Former smoker	229 (36)	19 (35)	0.991 (0.567–1.732)	0.974
Barthel Index before admission	48 ± 43	23 ± 33	0.987 (0.979–0.995)	0.001
Barthel Index on admission	25 ± 30	5 ± 16	0.964 (0.944–0.984)	< 0.001
Pneumococcal vaccination within 5 years	125 (20)	10 (19)	0.964 (0.485–1.915)	0.916
Chronic obstructive pulmonary disease	99 (16)	6 (11)	0.602 (0.240–1.512)	0.280
Chronic heart failure	119 (19)	16 (30)	1.699 (0.947–3.046)	0.075
Cerebrovascular diseases	217 (34)	16 (30)	0.749 (0.417–1.343)	0.332
Diabetes mellitus	98 (15)	10 (19)	1.256 (0.632–2.496)	0.515
Dementia	201 (32)	25 (46)	1.645 (0.963–2.810)	0.068
Gastroesophageal reflux	15 (2)	0 (0)	0.048 (0.000–124.317)	0.449
Malignancies	23 (4)	13 (13)	2.376 (1.017–5.551)	0.046
Corticosteroids	46 (7)	2 (4)	0.495 (0.120–2.030)	0.328
White blood cells, 10^3^/µL	10.7 ± 5.1	10.5 ± 5.9	1.000 (1.000–1.000)	0.856
Hemoglobin, g/dL	11.8 ± 4.7	10.6 ± 2.2	0.827 (0.737–0.928)	0.001
C-reactive protein, mg/dL	8.8 ± 7.3	10.2 ± 8.7	1.020 (0.986–1.054)	0.248
Aspartate aminotransferase, IU/L	31.4 ± 72.0	27.6 ± 23.0	0.997 (0.984–1.011)	0.706
Urea nitrogen, mg/dL	23.6 ± 14.3	32.6 ± 23.1	1.023 (1.011–1.035)	< 0.001
Creatinine, mg/dL	1.01 ± 0.82	1.31 ± 1.00	1.303 (1.063–1.596)	0.011
Albumin, g/dL	3.3 ± 2.1	2.7 ± 0.6	0.363 (0.255–0.517)	< 0.001
ESMcsa, cm^2^	20.0 ± 7.6	15.4 ± 4.9	0.999 (0.999–1.000)	< 0.001
ESMcsa/BSA	14.7 ± 4.9	11.7 ± 3.4	0.875 (0.819–0.936)	< 0.001
Inappropriate use of antibiotics	78 (12)	11 (20)	1.585 (0.817–3.076)	0.173

Data are presented as the number (%) or mean ± SD.

*BMI* body mass index, *BSA* body surface area, *CI* confidence interval, *ESM* erector spinae muscle, *ESMcsa* cross-sectional area of ESM, *HR* hazard ratio, *SD* standard deviation.

**Table 5:** Multivariate Cox proportional hazards analyses of 30-day in-hospital mortality.

	Model 1	p	Model 2	p
	HR (95% CI)		HR (95% CI)	
Female	0.421 (0.231–0.765)	0.005	0.424 (0.234–0.769)	0.005
Age, years	1.058 (1.014–1.104)	0.009	1.054 (1.010–1.100)	0.016
BMI, kg/m^2^	0.939 (0.866–1.019)	0.133	0.941 (0.868–1.020)	0.140
Loss of consciousness	1.961 (1.051–3.659)	0.034	1.734 (0.920–3.268)	0.088
Respiratory failure	3.501 (1.908–6.424)	< 0.001	3.181 (1.728–5.857)	< 0.001
Barthel Index before admission	0.993 (0.985–1.001)	0.092	–	–
Barthel Index on admission	–	–	0.979 (0.959–0.999)	0.041
Malignancies	2.600 (1.086–6.227)	0.032	2.558 (1.072–6.102)	0.034
ESMcsa/BSA	0.885 (0.819–0.956)	0.002	0.882 (0.816–0.953)	0.001

Data are presented as the number (%) or mean ± SD.

*BMI* body mass index, *BSA* body surface area, *CI* confidence interval, *ESM* erector spinae muscle, *ESMcsa* cross-sectional area of ESM, *HR* hazard ratio, *SD* standard deviation.

## Discussion

This study suggests that ESMcsa/BSA can independently predict in-hospital mortality and 30-day mortality in elderly patients with pneumonia. BMI, chronic heart failure, the Barthel Index, respiratory failure, hemoglobin levels and albumin levels, which were also identified as significantly different between survivors and non-survivors in the univariate analysis.

While BMI has been correlated with skeletal muscle size^20^, BMI is affected by both muscle and fatty tissue. This suggests that skeletal muscle size may be more sensitive than BMI as a measure of wasting and weakness. ESM is a major muscle of respiration along with the diaphragm and intercostal muscles^21^. If these respiratory muscles are weakened, breathing can be impaired, especially in exhalation^22^. A study investigating the relationship between ESM and mortality in patients with pulmonary *Mycobacterium avium complex* (MAC) suggested a relationship between ESMcsa and % predicted forced vital capacity^18^. The investigators prospectively included 260 patients who were diagnosed with MAC infection (21 patients died). Cox proportional hazards model analysis showed that the ESMcsa was independently associated with all-cause mortality (hazard ratio 2.76; 95% confidence interval 1.01–7.56; p = 0.047). However, the significance was lost when the % predicted functional vital capacity and forced expiratory volume in 1 s, were added to the model. This is also consistent with the results of a previous study focusing on patients with chronic obstructive pulmonary disease^10^. However, in many elderly patients with pneumonia, the testing of pulmonary function using a forced exhalation technique is not performed. Given that maximal inspiratory and expiratory pressures (MIPs and MEPs) do not require a forced exhalation maneuver and are thought to correlate with the strength of the diaphragm and intercostals, it would be interesting to measure MIPs and MEPs along with ESMcsa in a future prospective study.

A study assessing the impact of ESMcsa on activities of daily living (ADL) levels in patients with CAP showed that ESMcsa was associated with lower levels of ADL at the end of the treatment (hazard ratio 0.882; 95% confidence interval 0.806–0.946; p = 0.006), independent of age, sex, severity of pneumonia, nutritional status, or hydration status^23^. Besides its role as a respiratory muscle, the ESM also contributes to physical activities controlling the flexion of the vertebral column. This is consistent with the results we report here.

In Japan, most elderly patients with pneumonia have a chest CT on admission to hospital to evaluate lung involvement and rule out malignancy, parapneumonic effusion, or cardiac enlargement. Nevertheless, 34 patients (5%) had not undergone chest CT at admission posing a possible selection bias, as these patients may have been less sick and with typical clinical symptoms of acute pneumonia. Chest CT is not indicated for the routine evaluation of patients with CAP. We obtained Chest CT only in elderly patients who were thought to be at high risk for complex pneumonia or parapneumonic effusion and used these existing scans to evaluate the spinal erector muscle area; ESMcsa/BSA. We do not recommend the routine use of Chest CT scans for patients with CAP; however, when these scans are obtained, we show that the measurement of ESMcsa/BSA can provide valuable additional prognostic information.

Aspiration pneumonia more commonly occurs in patients with wasting, but the clinical presentation is usually similar to that of CAP not due to aspiration^4,24^. Thus, our study probably includes some patients with aspiration pneumonia. We did evaluate loss of consciousness, dementia and gastroesophageal reflex as known risk factors for aspiration but these were not significantly associated with mortality.

In conclusion, this study showed that ESMcsa/BSA independently predicted mortality in elderly patients with pneumonia. Both ADL levels and BMI, which are recognized as prognostic factors of pneumonia in elderly patients, were significantly correlated with ESMcsa/BSA. ESM contributes to both physical activities and respiratory functions. Therefore, ESMcsa/BSA may help predict in elderly patients with pneumonia. Decreased muscle mass may predispose to pneumonia and as well, can potentially make it more difficult for patients to recover from pneumonia.

## Methods

### Patients

This was a retrospective cohort study conducted at Tenshindo Hetsugi Hospital, a community hospital with 188 beds in Oita, Oita Prefecture, Japan. In this study, CAP was defined based on the criteria in the American Thoracic Society/Infectious Diseases Society of America guidelines^25^, using clinical signs and symptoms including cough, fever, and infiltrates on chest radiograph or chest CT. Patients with hospital‐acquired pneumonia, patients with underlying pulmonary diseases, and patients treated with immunosuppressants were excluded from this study. We included consecutive patients (age ≥ 65 years) admitted to the hospital between January 2015 and December 2017 for CAP, who had a chest CT in the 2 weeks before or after admission and whose height and weight were documented enabling the calculation of BMI and body surface area (BSA). The number of samples was calculated using EpiTools (https://epitools.ausvet.com.au/, two-tailed, α error = 0.05 and power = 0.8), and a total sample size of 687 patients was required according to a previous report that evaluated the association between ESMcsa and mortality^19^. The study protocol was approved by the Institutional Ethics Committee of Tenshindo Hetsugi Hospital (Approval Number: 13017; Approval Date: 4 April, 2019). Informed consent was waived by the Institutional Ethics Committee of Tenshindo Hetsugi Hospital because of the retrospective design of the study. This study was performed in accordance with relevant guidelines and regulations.

### Data collection and outcome

Patient data on admission included sex, age, body mass index (BMI), comorbidities, and laboratory data (i.e. white blood cell count, C-reactive protein levels, albumin levels, alanine transaminase) as routinely documented when a patient diagnosed with CAP is admitted. We evaluated daily physical activity before and on admission using the Barthel Index. The Barthel Index was introduced in 1965 and originally used a 0–20 scale^26^. It was modified by Granger et al. in 1979^27^ to include 0–10 points for each item (i.e., a total possible score of 0–100). Information relating to 10 basic activities of daily living are collected through the revised Barthel Index: bowels, bladder, grooming, toilet use, feeding, transfers, walking, dressing, climbing stairs, and bathing. We defined respiratory failure as < 90% SpO_2_ without supplemental oxygen inhalation on admission.

We set all-cause in-hospital and 30-day mortality as the primary outcomes. These data were extracted by respiratory physicians (TY and HY). Inappropriate antibiotics were defined as administration of antibiotics which did not cover pathogens identified in airway secretions.

### Evaluation of chest CT findings

A 320-detector rows CT scanner (AquilionONE; Toshiba Medical Systems, Tokyo, Japan) was used. Scans were obtained using 2.0-mm thick sections of contiguous images from the apex to the base of the lung. Images were captured at a window setting of − 600 HU (level) and 1500 HU (width). If the patient had undergone CT before referral to our hospital, we evaluated the CT features from the images captured at the referring institutes.

Chest CT images reconstructed using the mediastinal setting were used for the quantitative analysis of the ESM. By the evaluation method used in previous studies^28,29^, the area of the ESM on cross-sectional CT image (ESMcsa) was measured at the level of the lower margin of the 12th thoracic vertebra using a SYNAPSE volume analyzer (FUJIFILM Medical Co., Ltd., Tokyo, Japan). In brief, the left and right ESMs were identified and manually shaded, the cross-sectional areas of both ESMs were calculated, and the ESMcsa was presented as the sum of the right and left muscles. We calculated the difference between the maximum and minimum ESMcsa measured by three respiratory medicine specialists (with 11, 16, and 17 years of experience), who were blinded to clinical information. The measurement results were discussed when the difference between the maximum value and the minimum value differed from the mean by three times the standard deviation or more. ESMcsa was adjusted by the BSA (calculated using height and weight measurements).

### Statistical analysis

Statistical analyses were performed using the IBM SPSS statistics version 24 software package (IBM Japan, Tokyo, Japan). For two-tailed analyses, 95% confidence intervals were calculated. Variables with a p value < 0.05 in the univariate analysis were subsequently entered into the multivariate analysis. In order to explain how ESMcsa was independent of other variables as a predictor of in-hospital mortality and 30-day mortality, Cox proportional hazards regression analysis was used to evaluate the role of ESMcsa/BSA on in-hospital mortality after adjusting for other variables. Correlations between ESMcsa/BSA and other serial variables were analyzed using Pearson’s correlation coefficient (*r*) and Spearman’s rank correlation coefficient (ρ). For all analyses, a p-value < 0.05 denoted statistical significance.
