# Author Correction: Quantitative assessment of erector spinae muscles and prognosis in elderly patients with pneumonia

**DOI:** 10.1038/s41598-021-95677-1

**Published:** 2021-08-12

**Authors:** Hiroki Yoshikawa, Kosaku Komiya, Takashi Yamamoto, Naoko Fujita, Hiroaki Oka, Eiji Okabe, Mari Yamasue, Kenji Umeki, Bruce K. Rubin, Kazufumi Hiramatsu, Jun‑ichi Kadota

**Affiliations:** 1 Department of Respiratory Medicine and Infectious Diseases, Oita University Faculty of Medicine, 1‑1 Idaigaoka, Hasama‑machi, Yufu, Oita 879‑5593 Japan; 2 Department of Internal Medicine, Tenshindo Hetsugi Hospital, 5956 Nihongi, Nakahetsugi, Oita, Oita 879‑7761 Japan; 3 Department of Pediatrics, Virginia Commonwealth University School of Medicine, 1217 East Marshall Street, KMSB, Room 215, Richmond, VA 23298 USA; 4 Department of Medical Safety Management, Oita University Faculty of Medicine, 1‑1 Idaigaoka, Hasama‑machi, Yufu, Oita 879‑5593 Japan; 5 Nagasaki Harbor Medical Center, 6‑39 Shinchi‑machi, Nagasaki, 850‑8555 Japan

Correction to: *Scientific Reports* 10.1038/s41598-021-83995-3, published online 22 February 2021

The original version of this Article contained repeated errors in Tables 1, 2 and 4, where the values for “White blood cells, 10^3^/μL” and “ESMcsa, cm^2^” were incorrectly given. The correct and incorrect values appear below.

Table 1

Incorrect:

**Table:** 

Variables	All cases N = 689
ESMcsa, cm^2^	1965 ± 759

Correct:

**Table:** 

Variables	All cases N = 689
ESMcsa, cm^2^	19.7 ± 7.6

Table 2

Incorrect:

**Table:** 

	Survivor group n = 588	Non-survivor group n = 101	HR (95% CI)	p
White blood cells, 10^3^/µL	10,688 ± 5,013	10,877 ± 5,764	1.000 (1.000–1.000)	0.381
ESMcsa, cm^2^	2042 ± 763	1521 ± 558	0.999 (0.999–0.999)	< 0.001

Correct:

**Table:** 

	Survivor group n = 588	Non-survivor group n = 101	HR (95% CI)	p
White blood cells, 10^3^/µL	10.7 ± 5.0	10.9 ± 5.8	1.000 (1.000–1.000)	0.381
ESMcsa, cm^2^	20.4 ± 7.6	15.2 ± 5.6	0.999 (0.999–0.999)	< 0.001

Table 4

Incorrect:

**Table:** 

	Survivor group n = 635	Non-survivor group n = 54	HR (95% CI)	p
White blood cells, 10^3^/µL	10,733 ± 5,059	10,513 ± 5,892	1.000 (1.000–1.000)	0.856
ESMcsa, cm^2^	1998 ± 757	1540 ± 491	0.999 (0.999–1.000)	< 0.001

Correct:

**Table:** 

	Survivor group n = 635	Non-survivor group n = 54	HR (95% CI)	p
White blood cells, 10^3^/µL	10.7 ± 5.1	10.5 ± 5.9	1.000 (1.000–1.000)	0.856
ESMcsa, cm^2^	20.0 ± 7.6	15.4 ± 4.9	0.999 (0.999–1.000)	< 0.001

The original Article has been corrected.

